# Distinct Effects of Unfractionated Heparin versus Bivalirudin on Circulating Angiogenic Peptides

**DOI:** 10.1371/journal.pone.0034344

**Published:** 2012-04-11

**Authors:** Navin K. Kapur, Chetan Shenoy, Adil A. Yunis, Najwa N. Mohammad, Szuhuei Wilson, Vikram Paruchuri, Emily E. Mackey, Xiaoying Qiao, Ameer Shah, Michele L. Esposito, Richard H. Karas, Iris Z. Jaffe

**Affiliations:** 1 Molecular Cardiology Research Institute, Tufts Medical Center, Boston, Massachusetts, United States of America; 2 Division of Cardiology, Department of Medicine, Tufts Medical Center, Boston, Massachusetts, United States of America; University of Frankfurt - University Hospital Frankfurt, Germany

## Abstract

**Background:**

Human studies of therapeutic angiogenesis, stem-cell, and progenitor-cell therapy have failed to demonstrate consistent clinical benefit. Recent studies have shown that heparin increases circulating levels of anti-angiogenic peptides. Given the widely prevalent use of heparin in percutaneous and surgical procedures including those performed as part of studies examining the benefit of therapeutic angiogenesis and cell-based therapy, we compared the effects of unfractionated heparin (UFH) on angiogenic peptides with those of bivalirudin, a relatively newer anticoagulant whose effects on angiogenic peptides have not been studied.

**Methodology/Principal Findings:**

We measured soluble fms-like tyrosine kinase-1 (sFLT1), placental growth factor (PlGF), vascular endothelial growth factor (VEGF), and soluble Endoglin (sEng) serum levels by enzyme linked immunosorbent assays (ELISA) in 16 patients undergoing elective percutaneous coronary intervention. Compared to baseline values, sFLT1 and PlGF levels increased by 2629±313% and 253±54%, respectively, within 30 minutes of UFH therapy (p<0.01 for both; n = 8). VEGF levels decreased by 93.2±5% in patients treated with UFH (p<0.01 versus baseline). No change in sEng levels were observed after UFH therapy. No changes in sFLT1, PlGF, VEGF, or sEng levels were observed in any patients receiving bivalirudin (n = 8). To further explore the direct effect of anticoagulation on circulating angiogenic peptides, adult, male wild-type mice received venous injections of clinically dosed UFH or bivalirudin. Compared to saline controls, sFLT1 and PlGF levels increased by >500% (p<0.01, for both) and VEGF levels increased by 221±101% (p<0.05) 30 minutes after UFH treatment. Bivalirudin had no effect on peptide levels. To study the cellular origin of peptides after anticoagulant therapy, human coronary endothelial cells were treated with UFH and demonstrated increased sFLT1 and PlGF levels (ANOVA p<0.01 for both) with reduced VEGF levels (ANOVA p<0.05). Bivalirudin had no effect on peptide levels in vitro.

**Conclusions/Significance:**

Circulating levels of sFLT1, PlGF, and VEGF are significantly altered by UFH, while bivalirudin therapy has no effect. These findings may have significant implications for clinical studies of therapeutic angiogenesis, stem-cell and progenitor-cell therapy.

## Introduction

Since the discovery of vascular endothelial growth factor (VEGF), therapeutic angiogenesis, stem-cell and progenitor-cell therapy have attracted interest as an innovative approaches to manage end-stage ischemic coronary and peripheral artery disease [1], [2]. Although early, small-scale clinical studies demonstrated the benefits of pro-angiogenic and anti-angiogenic treatments, and underscored the potential effects of cyto-therapeutics largely by using bone marrow-derived cells, subsequent randomized-controlled trials have revealed mixed results with no large-scale data showing efficacy with respect to clinical outcomes [1], [2].

Heparin (unfractionated or low-molecular weight) is commonly used for the treatment of coronary and peripheral artery disease, especially in the settings of acute coronary syndromes (ACS) and percutaneous coronary intervention (PCI). Recent data have shown that heparin administration (in either unfractionated or low-molecular weight form) increases levels of soluble fms-like tyrosine kinase-1 (sFlt1) [3]–[6], a circulating splice variant of the VEGF receptor 1 that is expressed by endothelial cells, vascular smooth muscle cells, and monocytes [7], [8]. sFlt1 binds to pro-angiogenic factors including placental growth factor (PlGF), VEGF-A and VEGF-B and sequesters those ligands from acting on vascular cell surface receptors to promote angiogenesis and vascular health [9]. Endoglin (CD105) is another membrane-associated receptor that regulates the pro-angiogenic cytokine transforming growth factor beta (TGFβ1) [10]. The extracellular domain of endoglin can also be proteolytically cleaved and circulate as soluble Endoglin (sEng) [11]. Over the past decade, several lines of evidence have suggested that sEng may also negatively regulate angiogenesis [12]–[14].

Given the substantial use of heparin therapy in trials of therapeutic angiogenesis and stem- and progenitor-cell therapy, we compared the effects of unfractionated heparin (UFH) on circulating levels of sFlt1, sEng, VEGF_165_ and PlGF with those of a relatively new anticoagulant, bivalirudin, whose effects on angiogenic peptides have not been explored.

## Materials and Methods

### Patient Population

In this prospective observational study, we enrolled 16 human subjects presenting to Tufts Medical Center for elective PCI in the setting of chronic stable angina as defined by American College of Cardiology/American Heart Association guidelines [15]. All subjects had angiographically confirmed significant coronary artery disease, defined as a luminal narrowing greater than 70%, requiring PCI. All subjects had baseline Thrombolysis in Myocardial Infarction (TIMI) 3 flow in the coronary artery targeted for PCI. Other inclusion criteria were as follows: age >18 years and <90 years of age, sinus rhythm, and ability to provide informed consent. Patients with heparin or contrast allergy; hemodynamic or clinical instability; perceived interference with standard clinical care of patients; unsuccessful reperfusion, pregnancy, active or remote cancer, renal failure (estimated glomerular filtration rate <30 mL/min/1.73m^2^) or liver transaminases >2 times the upper limit of normal were excluded. All eligible subjects who agreed to enroll received either UFH or bivalirudin immediately before PCI as per standard dosing protocols. Specifically, UFH was administered as a 60 units (U)/kg bolus followed by a 10 U/kg/hour infusion. Bivalirudin was dosed with a 0.75 mg/kg bolus and a 1.75 mg/kg/hour continuous infusion. In all subjects, blood samples were obtained via the arterial sheath immediately before anticoagulant administration and at 30-, and 60- minutes after anticoagulation. In 3 patients, additional blood samples were obtained at 6 and 24 hours from the administration of the anticoagulant.

All physicians were blinded to the results of the serum analysis. Subjects received standard clinical care for elective PCI during their hospitalization, including electrocardiograms and cardiac biomarkers, and pharmacologic therapy including aspirin, clopidogrel, HMG-CoA reductase inhibitors, beta-blockers, and angiotensin converting enzyme-inhibitors, as clinically indicated. No subjects in this study required the use of glycoprotein IIb/IIIa inhibitors. Tufts Medical Center's Institutional Review Board approved this study. All subjects provided written informed consent.

#### Circulating Angiogenic Peptides *in vivo*

Sixteen-week-old, male, wild type C57/Bl6 mice received UFH or bivalirudin via tail-vein injection. Control groups included mice receiving normal saline or an inert polyanionic substance, dermatan sulfate (DSO4) via tail-vein injection (n = 4 per group). After 30 minutes of treatment, serum samples were obtained from the inferior vena cava.

#### Circulating Angiogenic Peptides *in vitro*

Human coronary artery endothelial cells (HCAEC; Cell Applications Inc., San Diego, CA, USA) were cultured to near confluence using normal humidified tissue culture incubators with 5% CO2. For anticoagulant stimulation studies, HCAECs were treated with UFH, bivalirudin, or DSO4 for 30 minutes. Conditioned media was then harvested for protein expression analysis.

#### Immunofluorescence

Human coronary artery endothelial cells (HCAECs) were grown in uncoated 6-well culture plates, rinsed in phosphate buffered saline (PBS) and fixed for 20 minutes in 4% formaldehyde in PBS. Fixed cells were treated with the anticoagulants using N-terminal- and C-terminal-specific Flt1 antibodies (C-17, sc-316, Santa Cruz Biotechnology, Santa Cruz, CA). Cellular fluorescence was imaged using a Zeiss LSM510 META confocal microscope system.

#### Biomarker Assays

Human blood samples were collected using serum separator tubes and allowed to clot for 30 minutes prior to centrifugation at 2000 g for 15 minutes. Serum samples were immediately stored at −80°C. sFlt1, sEng, VEGF_165_ and PlGF levels were measured in duplicate for each serum sample using commercially available human quantitative sandwich enzyme immunoassay kits (ELISA, R&D Systems, Minneapolis, MN, USA) according to the manufacturer's instructions. Time to completion of the human sFlt1 ELISA was 4.5 hours. For mouse serum analysis, sFlt1, sEng, VEGF_165_ and PlGF levels were measured using specific mouse ELISA kits (R&D Systems, Minneapolis, MN, USA). The ELISA kit for human VEGF used in our study detects only free VEGF, while the assay for mouse VEGF detects total VEGF, both bound and free. For *in vitro* sFlt1 analysis, conditioned media from incubated HCAECs were isolated, spun at 10,000 rpm for 5 minutes to remove cellular debris, and then analyzed using a human sFlt1 ELISA kit (R&D Systems, Minneapolis, MN, USA). Blinded observers performed all biomarker assays.

### Statistical Analysis

Data are expressed as mean±SE. Pair-wise comparisons were made using analysis of variance (ANOVA) for continuous variables. ANOVA with repeated measures (RM ANOVA) examined change in outcome variables over time. For *in vitro* data, 2-tailed t tests and 1-way ANOVA with a Bonferroni correction for inter-group comparisons were used. All statistical analyses were performed using SigmaStat Version 3.1 (Systat Software, Inc., Chicago, IL) and Statistical Package for the Social Sciences (SPSS, v16.0.1, SPSS, Inc., Chicago, IL). A P value of <0.05 was used to denote significant difference.

## Results

### Subject Characteristics

Characteristics of the study groups are provided in Table 1. The study groups were not different with respect to age, gender or race. No significant differences in medication use or baseline laboratory values were noted except for a higher number of patients receiving angiotensin converting enzyme-inhibitor therapy prior to PCI in the UFH group. All 16 subjects referred for elective PCI underwent single-vessel coronary intervention with no difference in the number of coronary stents deployed between the two groups (1.25±0.46 vs 1.625±0.74, UFH versus bivalirudin, p = NS) coronary stents. No intra-procedural complications occurred in any subject. For subjects receiving UFH, a mean body weight of 90 kg was observed, yielding a mean bolus infusion of 5400 U followed by a continuous infusion of 10 U/kg or 900 U/hr. The mean time for procedures was 1.3 hours; thus, on average, each patient received 6570 U of UFH total. For the bivalirudin group, mean body weight was 100 kg, leading to a bolus of 75 mg and 175 mg/hr infusion. The mean time for procedures was 1.2 hours; thus, on average, each patient received 110 mg of bivalirudin.

**Table 1:** Subject Characteristics.

Characteristic	Bivalirudin	Heparin	P value
	(n = 8)	(n = 8)	
**Clinical Characteristics**			
Age (years)	59.8±12.2	62.8±11.2	0.6
Sex (% men)	87.5	87.5	0.5
Race (% Caucasian)	87.5	100	0.2
Height (cm)	174.7±10	173.8±12.4	0.9
Weight (kg)	90±13.4	101.7±21.8	0.2
BSA (m2)	2.1±0.2	2.1±0.3	0.4
BMI	29.5±4.5	3.9±7.8	0.3
**Past Medical History (%)**			
Hypertension	87.5	87.5	0.5
Diabetes mellitus	12.5	50	0.1
Active tobacco use	25	0	0.1
Coronary artery disease	25	62.5	0.04
Hypercholesterolemia	87.5	87.5	0.5
Myocardial infarction	12.5	12.5	0.5
Cerebrovascular disease	0	0	N/A
Peripheral vascular disease	25	0	0.1
Atrial fibrillation	0	12.5	0.2
**Medications (%)**			
Aspirin	87.5	87.5	0.5
Clopidogrel	87.5	87.5	0.5
β-blocker	87.5	75	0.3
Angiotensin converting enzyme inhibitor	12.5	62.5	0.02
Calcium channel blocker	12.5	12.5	0.5
Angiotensin-receptor-blocker	25	0	0.1
Aldosterone antagonist	0	0	N/A
Diuretic	0	0	N/A
Anti-lipid agent	87.5	87.5	0.5
**Lab Values**			
Na+	139.6±2.9	137.9±0.8	0.07
K+	4.2±0.4	4.1±0.3	0.6
Cl-	106.1±3.5	103.8±0.9	0.06
CO2	26±1.7	26.9±1.2	0.3
BUN	14.5±3.0	14.6±6.6	0.9
Creatinine	1.0±0.4	0.9±0.3	0.9
Glucose	111.6±22.5	120.9±24.1	0.3
White blood cells	7.4±1.7	8.1±2.0	0.6
Hemoglobin	14.1±1.2	12.5±1.3	0.05
Platelets	185.8±22.8	188.6±44.9	0.9
Prothrombin time	12.4±0.7	12.8±0.7	0.5
PTT	22.5±2.8	30.5±13.2	0.2
INR	1.0±0.1	1.1±0.1	0.4
EF (%)	60.2±9.9	52.5±8.8	0.4

#### Differential Effects on Serum Angiogenic Peptide Levels of Heparin and Bivalirudin in Human Subjects

**Figure 1:**
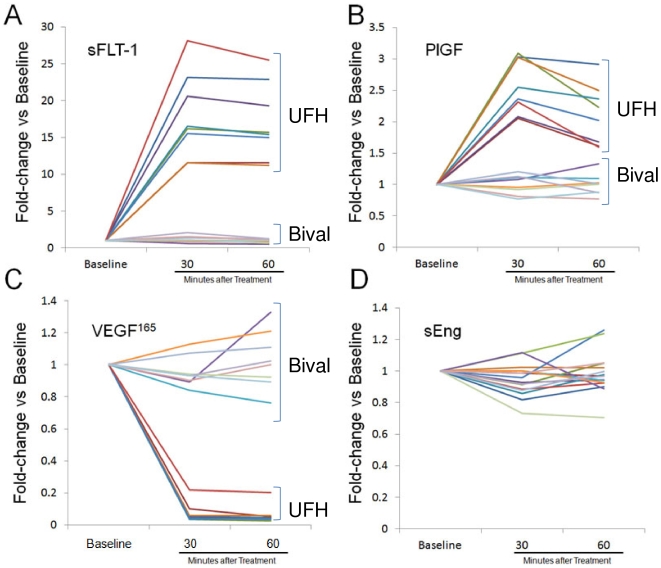
Effects on angiogenic peptides of UFH (dotted lines) and bivalirudin (dashed lines) in human subjects undergoing PCI. sFlt1 (A) and PlGF (B) were significantly increased by UFH but not bivalirudin, VEGF_165_ (C) was significantly decreased by UFH but not bivalirudin, and sEng (D) was unchanged with both UFH and bivalirudin.

Pre-treatment levels of measured angiogenic peptides were not different between the 2 groups (Figure 1). In patients receiving UFH, serum sFLT1 and PlGF levels were significantly increased within 30 minutes of UFH therapy (Figures 1A and 1B, respectively, p<0.001 versus baseline for both; n = 8). VEGF_165_ levels significantly decreased after 30 minutes of UFH therapy (Figure 1C, p<0.001 versus baseline). No significant changes in sEng levels were observed at 30 or 60 minutes after UFH therapy (Figure 1D). In patients receiving bivalirudin, no change in serum sFLT1, PGF, VEGF_165_ or sEng levels were observed. The assay for human VEGF used in our study detects only free VEGF. Therefore, any VEGF bound by heparin or by sFLT-1 will not be detected. We believe the significant reduction in circulating VEGF levels within 30 minutes of drug administration is due to the combined effect of heparin and sFLT-1 binding to human VEGF [16]. On the other hand, the assay for mouse VEGF detects total VEGF, BOTH bound and free. For this reason, we were not surprised to see that VEGF levels did not decrease. The increase in circulating VEGF may be due to release of VEGF from interstitial storage sites, such as skeletal muscle, by heparin treatment [17].

#### Changes in Serum Angiogenic Peptide Levels over 24 hours after Anticoagulant Therapy

**Figure 2:**
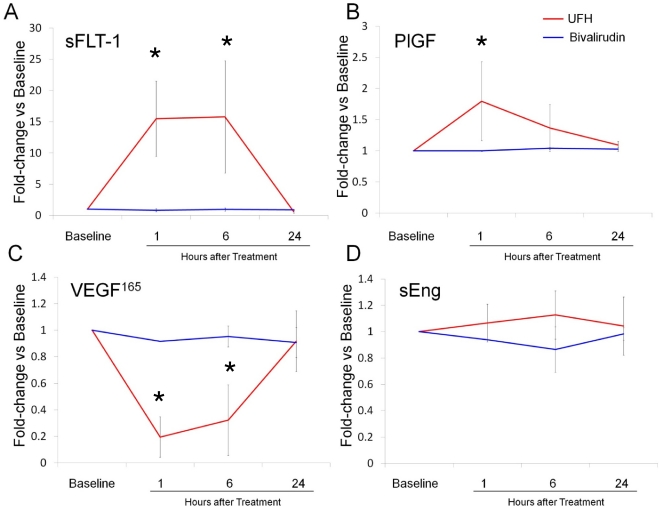
Time course of changes in sFlt1 (A), PlGF (B), VEGF_165_ (C) and sEng (D) with UFH and bivalirudin at baseline and 1, 6 and 24 hours after administration of anticoagulant. Note that levels of all angiogenic peptides revert to baseline levels by 24 hours with UFH and levels do not change significantly over 24 hours with bivalirudin.

To examine the time course of alterations in serum angiogenic peptides after anticoagulant therapy, serum was collected at the additional time-points of 6 and 24 hours after PCI-related anticoagulation in 6 patients. sFlt1 and PlGF levels returned to baseline by 24 hours after administration of UFH (Figure 2). VEGF_165_ levels were the lowest at 1 hour, and returned to baseline levels by 24 hours. sEng levels were not significantly changed up to 24 hours after UFH therapy. In patients receiving bivalirudin, no change in serum sFLT1, PlGF, VEGF_165_ or sEng levels were observed up to 24 hours after administration.

#### Differential Effects on Serum Angiogenic Peptide Levels of Heparin and Bivalirudin *in vivo* in wild type C57/Bl6 mice

**Figure 3:**
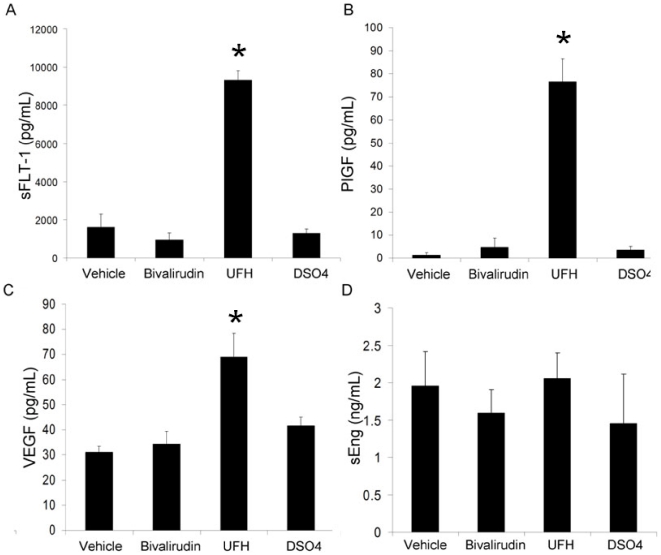
Effects on angiogenic peptide levels of UFH and bivalirudin *in vivo* in wild type C57/Bl6 mice. Treatment with UFH for 30 minutes resulted in a rapid and significant increase in mouse serum sFlt1 (A) and PGF (B) levels (>500% increase, p<0.01, for both) compared to normal saline vehicle. UFH also produced a modest but significant increase in mouse VEGF_165_ (C) levels (221+101%, p<0.05). Treatment with DSO4 did not influence sFlt1 or PlGF levels. VEGF_165_ levels were modestly increased by DSO4 treatment. Bivalirudin treatment had no effect on sFlt1, PlGF, and VEGF_165_ levels. sEng (D) levels were mildly increased with vehicle, UFH, bivalirudin and DSO4, with no significant differences between the 4 treatments.

To explore the effects of UFH and bivalirudin in the absence of atherosclerotic vascular disease and potential ischemia, these anticoagulants were tested in a mouse model. As in our human subjects, treatment with UFH for 30 minutes resulted in a rapid and significant increase in mouse serum sFlt1 and PGF levels (Figures 3A and 3B respectively, >500% increase, p<0.01, for both) compared to normal saline controls (NS). UFH also produced a modest but significant increase in mouse VEGF_165_ levels (Figure 3C, 221+101%, p<0.05). Treatment with DSO4 did not influence sFlt1 or PlGF levels. VEGF_165_ levels were modestly increased by DSO4 treatment. Bivalirudin treatment had no effect on sFlt1, PlGF, and VEGF_165_ levels in the mouse. These data support that the differential effects of UFH and bivalirudin on serum angiogenic peptide levels are independent of the presence of atherosclerotic disease.

#### Differential effects of Anticoagulants on Angiogenic Peptide Release from Human Coronary Artery Endothelial Cells

**Figure 4:**
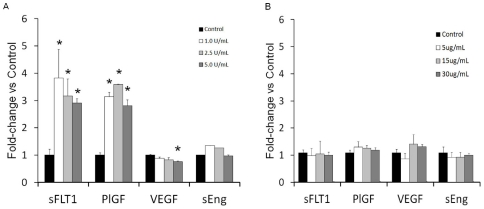
Levels of angiogenic peptides from human coronary artery endothelial cells treated with unfractionated heparin or bivalirudin. A) Conditioned media levels of sFlt1, PlGF, VEGF and sEng 30 minutes after treatment with increasing concentrations of UFH. (p<0.05 vs control). B) Conditioned media levels of sFlt1, PlGF, VEGF and sEng 30 minutes after treatment with increasing concentrations of bivalirudin. No differences were observed after treatment with bivalirudin.

Endothelial cells are known to express sFlt1, PlGF, VEGF, and sEng. To explore the endothelium as a potential source of circulating peptides we stimulated HCAECs with various concentrations of UFH and bivalirudin. Conditioned media levels of sFLT1 and PlGF were significantly increased and VEGF_165_ levels reduced 30 minutes after treatment of HCAECs with UFH (Figure 4A). These data are consistent with our clinical observations and support the likelihood that these peptides are released from the endothelium after treatment with UFH.

**Figure 5:**
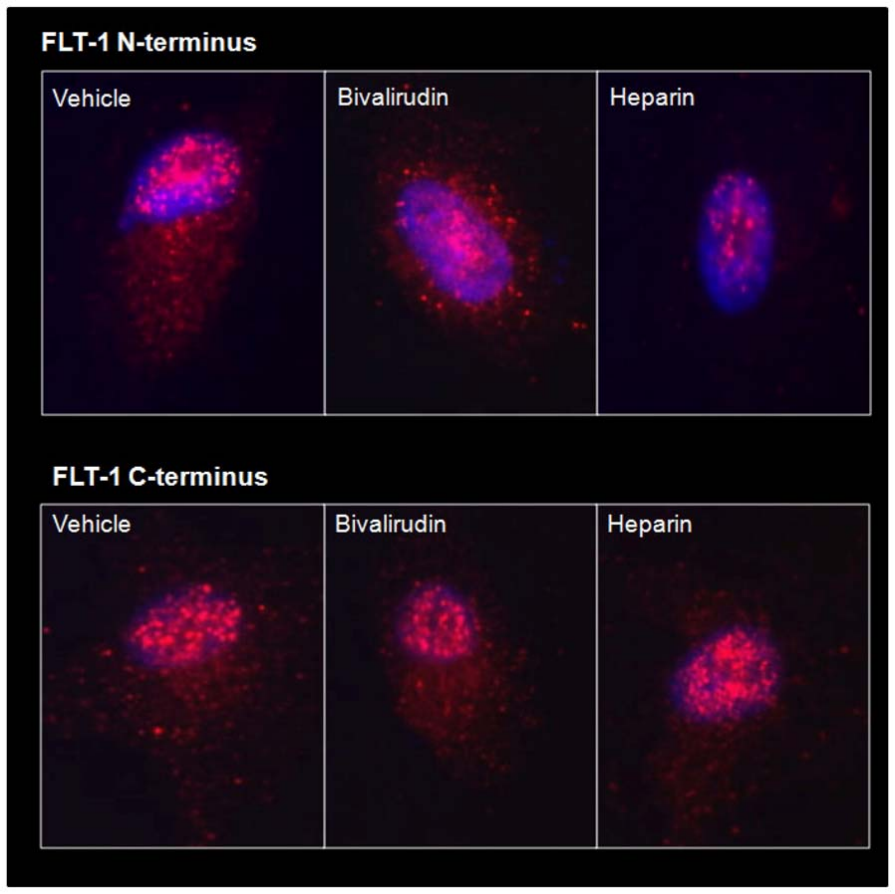
Effects of heparin and bivalirudin on angiogenic peptide release from HCAECs. Immunofluorescent microscopy shows a significant reduction in cell surface expression of the sFlt1 N-terminal extracellular domain (top panel) with no change in C-terminal expression (bottom panel). Treatment with bivalirudin had no effect on N-terminal or C-terminal expression.

Since levels of sFLT1 are rapidly increased by UFH stimulation, we next examined whether sFLT1 release from HCAECs is due to cleavage from the cell surface. To test this hypothesis, immunofluorescence microscopy was performed on anticoagulant-treated HCAECs using an N-terminal- and a C-terminal-specific Flt1 antibody. Within 30 minutes of stimulation with UFH, we observed a significant reduction in cell surface expression of the sFlt1 N-terminal extracellular domain with no change in C-terminal expression (Figure 5).

Treatment with bivalirudin had no effect on N-terminal or C-terminal expression (Figure 5).

## Discussion

The primary finding of our study is that administration of UFH results in significant changes in circulating levels of angiogenic peptides, while bivalirudin does not. sFlt1 and PlGF were increased, while VEGF_165_ levels were decreased with UFH. We demonstrated these differences *in vivo* using human subjects undergoing PCI for coronary artery disease and healthy C57/Bl6 mice, and *in vitro* using HCAECs. Since we recently demonstrated that sFlt1 levels are elevated early after an acute coronary occlusion [18], we used only patients with chronic stable angina and also showed that the differential effects of UFH and bivalirudin on serum angiogenic peptide levels are independent of the presence of atherosclerotic disease.

The underlying mechanisms for the increase in sFlt1 levels after heparin therapy are currently under investigation, although it is believed that sFlt1, which is bound to heparan sulfate proteoglycans, is released with heparin treatment because of competitive binding at its heparin-binding site [4]. The binding domain for heparin has been previously characterized on the most potent VEGF splice variant, VEGF_165_ [19]. The decrease in circulating VEGF_165_ levels after administration of UFH is potentially due to sequestration of VEGF into the extracellular matrix. We did observe a more dramatic reduction in VEGF levels after UFH treatment in patients as compared to HCAECs *in vitro*. There are several potential explanations for this difference. First, endothelial cells may not be the only source of circulating VEGF. Second, the *in vitro* model does not fully recapitulate the effect of circulating blood. Dissociation of VEGF from UFH or sFLT1 may occur *in vitro* and limit the amount of free VEGF detected by the ELISA kit, whereas the kinetics of UFH/VEGF or sFLT1/VEGF binding in circulating blood may be different. Third, once heparin or sFLT1 binds to VEGF *in vivo*, these moieties may be sequestered by the interstitium, which is not recapitulated by the *in vitro* system.

Therapeutic angiogenesis depends on the ability of angiogenic factors to induce the formation of a collateral blood supply, effectively bypassing a diseased coronary or peripheral artery. Over 25 phase II or phase III clinical trials involving approximately 2500 patients with coronary and peripheral artery disease, have assessed the efficacy of angiogenic cytokine protein and gene therapy involving VEGFs, fibroblast growth factors (FGFs) and other factors, with largely disappointing results showing no substantial benefit of therapeutic angiogenesis for cardiovascular disease [1]. Similarly, stem-cell and progenitor cell-based therapy is proposed to repair ischemic and injured cardiac tissue through mechanisms that include vascular repair, vasculogenesis and angiogenesis [2]. Angiogenic growth factors and stem- and progenitor-cells are frequently delivered via surgical or percutaneous procedures – either intracoronary or percutaneous intramyocardial injections – that often necessitate anticoagulant therapy. Our findings suggest that the use of UFH in this setting may limit the clinical utility of pro-angiogenic factors. Furthermore, the use of UFH may confound the ability to use angiogenic peptides as clinical biomarkers.

The antiangiogenic properties of heparin in cancer have been known for nearly 3 decades [20]. Heparin has previously been shown to influence the angiogenic response to basic fibroblast growth factor (bFGF) and other proangiogenic factors in cancer [21]. Our findings of elevated sFlt1 levels with heparin raise the possibility that heparin administration during procedures for delivery of angiogenic growth factors and stem- and progenitor-cells was anti-angiogenic, potentially contributing to the negative results of clinical trials.

Bivalirudin is a relatively new antithrombotic therapy, which acts through dual inhibition of thrombin and collagen-dependent platelet activation [22]. Bivalirudin has been shown to improve bleeding outcomes and short-term outcomes when compared with heparin [23], [24]. Based on our findings that bivalirudin has no effect on circulating angiogenic peptides, we propose that bivalirudin be used as the anticoagulant during procedures for delivery of angiogenic growth factors and stem- and progenitor-cells in ongoing and future trials. Trials with negative results that involved the use of heparin may need to be revisited in light of our findings. Bivalirudin should also be preferred in the setting of angiogenic therapy early during an acute myocardial infarction, at which time sFlt1 levels are significantly elevated [18].

The choice of anticoagulant therapy may also carry significant implications in other patient groups that require long-term anticoagulation and in whom angiogenesis and sFlt1 levels are relevant, such as cancer patients with venous thromboembolism, pregnant women with premature labor, and patients undergoing hemodialysis. Further research is necessary to understand all the clinical ramifications of the potential anti-angiogenic properties of heparin therapy.

## References

[1] Zachary I, Morgan RD (2011). Therapeutic angiogenesis for cardiovascular disease: biological context, challenges, prospects.. Heart.

[2] Tongers J, Losordo DW, Landmesser U (2011). Stem and progenitor cell-based therapy in ischaemic heart disease: promise, uncertainties, and challenges.. Eur Heart J.

[3] Sela S, Natanson-Yaron S, Zcharia E, Vlodavsky I, Yagel S (2011). Local retention versus systemic release of soluble VEGF receptor-1 are mediated by heparin-binding and regulated by heparanase.. Circ Res.

[4] Searle J, Mockel M, Gwosc S, Datwyler SA, Qadri F (2011). Heparin Strongly Induces Soluble fms-Like Tyrosine Kinase 1 Release In Vivo and In Vitro.. Arterioscler Thromb Vasc Biol.

[5] Carroll TY, Mulla MJ, Han CS, Brosens JJ, Chamley LW (2011). Modulation of trophoblast angiogenic factor secretion by antiphospholipid antibodies is not reversed by heparin.. Am J Reprod Immunol.

[6] Wong NS, Buckman RA, Clemons M, Verma S, Dent S (2010). Phase I/II trial of metronomic chemotherapy with daily dalteparin and cyclophosphamide, twice-weekly methotrexate, and daily prednisone as therapy for metastatic breast cancer using vascular endothelial growth factor and soluble vascular endothelial growth factor receptor levels as markers of response.. J Clin Oncol.

[7] Kendall RL, Wang G, DiSalvo J, Thomas KA (1994). Specificity of vascular endothelial cell growth factor receptor ligand binding domains.. Biochem Biophys Res Commun.

[8] Matthews W, Jordan CT, Wiegand GW, Pardoll D, Lemischka IR (1991). A receptor tyrosine kinase specific to hematopoietic stem and progenitor cell-enriched populations.. Cell.

[9] Maglione D, Guerriero V, Viglietto G, Delli-Bovi P, Persico MG (1991). Isolation of a human placenta cDNA coding for a protein related to the vascular permeability factor.. Proc Natl Acad Sci U S A.

[10] Cheifetz S, Bellon T, Cales C, Vera S, Bernabeu C (1992). Endoglin is a component of the transforming growth factor-beta receptor system in human endothelial cells.. J Biol Chem.

[11] Venkatesha S, Toporsian M, Lam C, Hanai J, Mammoto T (2006). Soluble endoglin contributes to the pathogenesis of preeclampsia.. Nat Med.

[12] Castonguay R, Werner ED, Matthews RG, Presman E, Mulivor AW (2011). Soluble endoglin specifically binds bone morphogenetic proteins 9 and 10 via its orphan domain, inhibits blood vessel formation, and suppresses tumor growth.. J Biol Chem.

[13] Levine RJ, Lam C, Qian C, Yu KF, Maynard SE (2006). Soluble endoglin and other circulating antiangiogenic factors in preeclampsia.. N Engl J Med.

[14] Lopez-Novoa JM, Bernabeu C (2010). The physiological role of endoglin in the cardiovascular system.. Am J Physiol Heart Circ Physiol.

[15] Gibbons RJ, Chatterjee K, Daley J, Douglas JS, Fihn SD (1999). ACC/AHA/ACP-ASIM guidelines for the management of patients with chronic stable angina: a report of the American College of Cardiology/American Heart Association Task Force on Practice Guidelines (Committee on Management of Patients With Chronic Stable Angina).. J Am Coll Cardiol.

[16] Maynard SE, Min JY, Merchan J, Lim KH, Li J (2003). Excess placental soluble fms-like tyrosine kinase 1 (sFlt1) may contribute to endothelial dysfunction, hypertension, and proteinuria in preeclampsia.. J Clin Invest.

[17] Kut C, Mac Gabhann F, Popel AS (2007). Where is VEGF in the body? A meta-analysis of VEGF distribution in cancer.. Br J Cancer.

[18] Kapur NK, Heffernan KS, Yunis AA, Nguyen TA, Aronovitz MJ (2011). Elevated soluble fms-like tyrosine kinase-1 levels in acute coronary occlusion.. Arterioscler Thromb Vasc Biol.

[19] Neufeld G, Cohen T, Gengrinovitch S, Poltorak Z (1999). Vascular endothelial growth factor (VEGF) and its receptors.. FASEB J.

[20] Folkman J, Langer R, Linhardt RJ, Haudenschild C, Taylor S (1983). Angiogenesis inhibition and tumor regression caused by heparin or a heparin fragment in the presence of cortisone.. Science.

[21] Rak J, Weitz JI (2003). Heparin and angiogenesis: size matters!. Arterioscler Thromb Vasc Biol.

[22] Kimmelstiel C, Zhang P, Kapur NK, Weintraub A, Krishnamurthy B (2011). Bivalirudin is a dual inhibitor of thrombin and collagen-dependent platelet activation in patients undergoing percutaneous coronary intervention.. Circ Cardiovasc Interv.

[23] Kastrati A, Neumann FJ, Mehilli J, Byrne RA, Iijima R (2008). Bivalirudin versus unfractionated heparin during percutaneous coronary intervention.. N Engl J Med.

[24] Rassen JA, Mittleman MA, Glynn RJ, Alan Brookhart M, Schneeweiss S (2010). Safety and effectiveness of bivalirudin in routine care of patients undergoing percutaneous coronary intervention.. Eur Heart J.

